# Associations of the COVID-19 pandemic with the economic status and mental health of people affected by the Fukushima disaster using the difference-in-differences method: The Fukushima Health Management Survey

**DOI:** 10.1016/j.ssmph.2021.100801

**Published:** 2021-04-15

**Authors:** Michio Murakami, Tomoyuki Kobayashi, Yuichi Oikawa, Saori Goto, Maho Momoi, Yoshitake Takebayashi, Tetsuya Ohira, Seiji Yasumura, Masaharu Maeda

**Affiliations:** a Radiation Medical Science Center for the Fukushima Health Management Survey, Fukushima Medical University, 1 Hikarigaoka, Fukushima, 960-1295, Japan; b Department of Health Risk Communication, Fukushima Medical University School of Medicine, 1 Hikarigaoka, Fukushima, 960-1295, Japan; c Department of Disaster Psychiatry, Fukushima Medical University School of Medicine, 1 Hikarigaoka, Fukushima, 960-1295, Japan; d Department of Epidemiology, Fukushima Medical University School of Medicine, 1 Hikarigaoka, Fukushima, 960-1295, Japan; e Department of Public Health, Fukushima Medical University School of Medicine, 1 Hikarigaoka, Fukushima, 960-1295, Japan

**Keywords:** Alcohol-related disorders, COVID-19, Difference-in-differences method, Fukushima nuclear accident, Psychological distress, Sleep

## Abstract

Although the coronavirus disease 2019 (COVID-19) pandemic and relevant preventive measures can affect the economic status and mental health of the public, their effect remains unraveled owing to a limited number of surveys conducted before and during the COVID-19 pandemic. We investigated the association of COVID-19 and relevant measures with multivariate outcomes among people affected by the Fukushima disaster in 2011 using the difference-in-differences (DID) method. We then analyzed the associations between sociodemographic factors and outcomes. We assessed psychological distress, problem drinking, insomnia state, unemployment, household economic decline, and interpersonal problems using three questionnaire surveys administered in 2018, 2019, and 2020. Participants were grouped according to three time periods by dates of voluntary stay-at-home requests (February 26) and the declaration of emergency (April 16) in Japan. The years 2020 and 2019 were regarded as the treatment group and control group, respectively, after confirming that no DIDs were found between 2018 and 2019. We performed regression analyses to identify the risk factors for outcomes. The DIDs were significant for household economic decline after the declaration of emergency, whereas problem drinking significantly improved. No significant DIDs were observed for other mental health outcomes including psychological distress and insomnia state. Absence of counselors was positively and significantly associated with all outcomes in 2020. Overall, people affected by the Fukushima disaster experienced more economic damage after the declaration of emergency during the COVID-19 pandemic but their mental health status did not reduce. Identifying people who have no counselors and providing them with support are emergent requirements to prevent a subsequent mental health decline.

## Highlights

• The economic status and mental health were evaluated after coronavirus disease 2019.
• We assess the effect in Fukushima residents by the difference-in-differences method.
• The economic status worsened after governmental restrictions of social activities.
• The mental health status was maintained after implementing public measures.
• Lack of counselors was a risk factor for a worsening economy and poor mental health.

## 1 Introduction

The coronavirus disease 2019 (COVID-19) pandemic has resulted in considerable loss of human health and lives. As of April 11, 2021, the number of confirmed COVID-19 cases and deaths exceeded 134 million and 2.9 million, respectively (World Health Organization, 2021). Public preventive measures including physical distancing, stay-at-home, and lockdown of cities have been implemented globally to prevent the spread of the disease (Agarwal & Sunitha, 2020). Consequently, these measures might result in increasing concerns about worsening economic status and psychological health, including psychological distress, problem drinking, and sleep-related problems (Altena et al., 2020; Da, Im, & Schiano, 2020; Qian & Fan, 2020; Rajkumar, 2020). Although a decline in mental health status such as psychological distress has often been reported among COVID-19 patients, health professionals, and the general population, there are very limited studies to clarify the effect of the COVID-19 pandemic and relevant preventive measures on the economic status and psychological health of the public, and most studies have a cross-sectional study design (Krishnamoorthy et al., 2020; Xiong et al., 2020). This warrants surveys to be conducted before and during the COVID-19 pandemic and implementation of the associated public measures.

After the spread of COVID-19, the Japanese central government implemented a voluntary stay-at-home measure on February 26, 2020, and declared the state of emergency in all prefectures, including Fukushima Prefecture, on April 16, 2020 (Table S1 in the Supplementary Materials). The declaration of the state of emergency was reduced to eight prefectures (Hokkaido, Saitama, Chiba, Tokyo, Kanagawa, Kyoto, Osaka, and Hyogo) on May 14, 2020, five prefectures (Hokkaido, Saitama, Chiba, Tokyo, and Kanagawa) on May 21, 2020, and completely lifted on May 25, 2020. The declaration of the state of emergency in Japan does not have a legal binding force on an individual's behavior, such as lockdown of cities in other countries, but the emergency aims at strongly restricting social activities such as school, business, and outside movement/travel. Consequently, social activities in Japan have sharply dropped after the declaration of the state of emergency, as evidenced in terms of visit frequency in recreation, workplaces, and transit stations (Parady et al., 2020).

The Fukushima Health Management Survey (FHMS), which was commissioned by Fukushima Prefecture, has been conducted to monitor and support the health of residents in Fukushima; it has a large cohort study design after the study on the Great East Japan Earthquake and Fukushima nuclear accident in March 2011 (hereinafter, “Fukushima disaster”) (Yasumura et al., 2012). As a part of the FHMS, a mental health and lifestyle survey questionnaire was sent to approximately 210,000 people affected by the Fukushima disaster at the end of January or the beginning of February every year since 2012. The survey conducted in 2020 therefore included participants who responded to the questionnaire before and after the implementation of the Japanese central governmental measures of voluntary stay-at-home on February 26, 2020, and the declaration of the state of emergency in all the prefectures on April 16. This condition allows us to apply the difference-in-differences (DID) design (Puhani, 2012), to quantify the association of public measures related to COVID-19 with the economic status and psychological health among the survey participants. Multivariate outcomes regarding economic status and psychological health can also be assessed, as the survey questionnaire includes psychological distress, problem drinking, insomnia state, and life events experienced within a year (e.g., unemployment, household economic decline, and interpersonal problems). The survey also serves to provide brief documental or telephone support for high-risk individuals among participants of the survey through identification on the basis of the questionnaire responses. Details of support were described elsewhere (Momoi, Murakami, Horikoshi, & Maeda, 2020). Furthermore, the survey is expected to reveal risk factors for these outcomes among sociodemographic factors to develop public measures to support high-risk people.

This study had two objectives. First, we assessed the association of public measures related to COVID-19 with multivariate outcomes related to the economic status and mental health of the people affected by the Fukushima disaster in Japan, using the DID method. Second, we investigated the associations between sociodemographic factors (i.e., age, sex, location, and absence of counselors) and the outcomes.

## 2 Methods

### 2.1 Study design

The DID method requires a treatment group and a control group in two time periods (Card & Krueger, 1994). The population mean is estimated from the measured values of those who participated in the survey (sample). The data are not necessarily analyzed as individual linked data but as a comparison of the control group and the treatment group before and after a certain intervention (Puhani, 2012). However, the COVID-19 pandemic and relevant public preventive measures such as governmental measures have effects on all people. We therefore set participants of the survey in 2019 and 2020 as the control group and the treatment group, respectively.

Regarding the mental health and lifestyle survey in 2020, the questionnaires were distributed from January 30, 2020 to February 14, 2020, and the data obtained from the responses by May 21, 2020 were used. We set May 21, 2020 as the end date of our survey because, this date came after the state of emergency in Fukushima Prefecture was lifted (May 14, 2020) but before it was lifted in all areas in Japan (May 25, 2020). We grouped the participants into three time periods based on the date that the data were collected: Time 1, start of the questionnaire distribution up to February 25; Time 2, from February 26 to April 15; Time 3, from April 16 to May 21 (all the dates inclusive). It should be noted that the first case of COVID-19 in Fukushima Prefecture was confirmed on March 7, 2020 (Fukushima Prefecture, 2020).

We assessed the difference between two years (2020 *vs.* 2019) in differences among time periods (Time 2 or 3 *vs.* Time 1) for the outcomes. The participants of the survey in 2018 were assessed to confirm whether there were no differences between 2018 and 2019 in differences among time periods.

As there were no differences in the population across years, we used the data of the participants who responded to the survey at least once. If only those who responded in all three years were included in the analyses, the sample would be biased toward such a group. In addition, the number of eligible participants would be reduced further, whereas the number of Time 3 participants was 242–820 in the case that those who responded at least once were included (Table S2). For these reasons, we did not include only those participants who responded in all three years.

Note that the respondents were different before and after the intervention (Time 1 and Time 2 or Time3). Ideally, using the same participants before and after the intervention is preferable. Our approach could yield different characteristics among the participants who responded earlier and later. This means that there could be a bias associated with the time of responses because of interest in the survey or other factors. Instead, this study mitigates such an effect by taking the difference between 2019 (control group) and 2020 (treatment group). We also adjust for covariates, such as age, to reduce the bias.

### 2.2 Participants

The questionnaires were distributed to residents who lived in 13 municipalities spanning evacuation order areas during the Fukushima disaster or the time of the surveys: Hirono Town, Naraha Town, Tomioka Town, Kawauchi Village, Okuma Town, Futaba Town, Namie Town, Katsurao Village, Iitate Village, Minamisoma City, Tamura City, Kawamata Town, and hotspot areas in Date City. The distribution of questionnaires for each year was done from February 1 to 26, 2018; January 31, 2019 to February 15, 2019; and January 30, 2020 to February 14, 2020. The number of adult respondents was 36489 (response rate: 20%) in 2018, 36079 (20%) in 2019, and 34038 (19%) in 2020. We excluded participants whose responses were collected on and after May 22 of the survey year or who did not respond to the questionnaire on their own. The total number of participants was 32304 in 2018, 31940 in 2019, and 30527 in 2020 (Fig. S1).

### 2.3 Outcomes

We investigated six outcomes: psychological distress, problem drinking, insomnia state, unemployment, household economic decline, and interpersonal problems.

Psychological distress was measured using the Kessler 6-item scale (K6) (Kessler et al., 2003). K6 consists of six brief questions about non-specific mental health distress during the past 30 days, with the total scores in the range of 0–24. We used a cutoff score of K6 ≥ 13 as an indicator of serious mental illness (Kessler et al., 2003) for consistency with other studies (Suzuki et al., 2015; Ueda et al., 2019).

Problem drinking was assessed using the CAGE (Cut down, Annoyed, Guilty, and Eye-opener) questionnaire (Ewing, 1984) among those whose age was ≥20 years and who answered current drinking frequency. Problem drinking was defined as participants who answered “drink (one or more times a month)” as current drinking frequency and whose CAGE scores were ≥2 (Ewing, 1984; Ueda et al., 2019).

An insomnia state was measured using the simplified Japanese version of the Athens Insomnia Scale (AIS-SJ) (Iwasa et al., 2019). The AIS-SJ scores range from 0 to 8 and were treated as continuous variables, in accordance with a previous study (Iwasa et al., 2019).

Regarding life events experienced within a year, three outcomes were evaluated: unemployment, household economic decline, and interpersonal problems. We used a questionnaire item that allows the participants to select multiple responses.

We also used sociodemographic factors including age, sex, location (i.e., inside/outside of Fukushima Prefecture), and presence/absence of counselors (e.g., family and relatives; friend/acquaintance; coworker/supervisor; municipal consultation service; prefectural consultation counter; mental health welfare center; Fukushima Center for Disaster Mental Health; home-visit nursing and care service organizations; medical institutions such as psychosomatic medicine, psychiatry, neurology, and mental health clinics; medical institutions other than those listed above; religious organizations; other), because these factors were associated with mental health (Suzuki et al., 2015; Ueda et al., 2019) and would be useful in developing public measures to support high-risk people. Age was set based on the date when the distribution of the questionnaires started. The characteristics of the participants are summarized in Table S2.

### 2.4 Statistical analysis

Multiple linear regression analysis was used as the DID model because the linear model based on the interaction term can appropriately estimate the DID (Puhani, 2012). Linear models were used for continuous variables (AIS-SJ) and binary outcomes (other variables). Robust standard errors were calculated. In this study, we used the interaction term between time periods and years. Dummy variables were created by setting Time 1 and the year 2019 at references. The analyses were separately performed for the datasets of 2018 *vs.* 2019 or of 2020 *vs.* 2019. We included sociodemographic factors as covariates in the models. The missing data were not included in the analyses. The number of participants included in the analyses ranged from 46,012 to 51,877, corresponding to 72–81% of the total number of participants. As a sensitivity analysis, we also performed a regression model for all the data from 2019 to 2020 or 2018 and 2019, with the same outcomes and covariates, by using a six-category exposure variable (three time periods in two years) instead of the DID method. Time 3 in 2019 was used as a reference.

We then separately performed multiple regression analyses for the datasets of 2019 or 2020. We used linear regression analysis for AIS-SJ scores and binary logistic regression analyses for other outcomes. Time periods and sociodemographic factors were included as explanatory variables. The number of participants included in the analyses ranged from 22,991 to 25,593, corresponding to 72–82% of the total number of participants.

All analyses were performed using IBM SPSS Statistics 24.

## 3 Results

Participants in 2020 were mostly collected at Time 1 (69%), followed by Time 2 (30%) and Time 3 (1%) (Fig. S1 and Table S2). A similar trend was found for the year 2019 (i.e., Time 1, 64%; Time 2, 35%; Time 3, 1%), while the proportion in Time 2 was abundant in 2018 (i.e., Time 1, 29%; Time 2, 68%; Time 3, 3%).

Comparisons between 2018 and 2019 showed no significant DIDs for all outcomes except psychological distress between Time 2 and Time 1 and unemployment between Time 3 and Time 1 (Table 1). Comparisons between 2020 and 2019 showed significant positive DIDs for unemployment and household economic decline between Time 3 and Time 1. The unstandardized regression coefficient B (95% confidence interval [CI]) was 0.041 (0.014–0.069) for unemployment and 0.075 (0.016–0.134) for household economic decline. The significant and negative DID was observed for problem drinking between Time 3 and Time 1: −0.053 (−0.098 to −0.007). No significant DIDs were observed for psychological distress, AIS-SJ, or interpersonal problems. The results of other variables are summarized in Table S3. A sensitivity analysis using 2019 Time 3 as a reference also showed a significantly positive association with unemployment in all time categories and household economic decline in 2020 Time 3, and a significantly negative association with problem drinking in 2020 Time 3 (Table S4).

**Table 1:** Difference-in-differences (DID) of outcomes. (a) 2018 vs. 2019, (b) 2020 vs. 2019. B: unstandardized regression coefficient, CI: confidence interval.

	Psychological distress		Problem drinking		AIS-SJ^a^		Unemployment		Household economic decline		Interpersonal problems	
	B (95%CI)	P	B (95%CI)	P	B (95%CI)	P	B (95%CI)	P	B (95%CI)	P	B (95%CI)	P
(a) 2018 vs 2019												
N	51877		50735		46539		50419		50423		50409	
DID (ref = 2019, Time 1)												
2018, Time 2	0.010 (0.001–0.018)	0.026	−0.007 (−0.016–0.002)	0.123	0.051 (−0.034–0.135)	0.239	0.005 (−0.002–0.012)	0.189	−0.007 (−0.019–0.005)	0.257	0.002 (−0.007–0.011)	0.682
2018, Time 3	−0.005 (−0.044–0.034)	0.802	−0.035 (−0.079–0.009)	0.120	0.000 (−0.334–0.334)	0.998	0.025 (0.003–0.048)	0.026	0.024 (−0.026–0.075)	0.343	−0.006 (−0.050–0.038)	0.796
(b) 2020 vs 2019												
N	50746		49660		46012		50065		50070		50066	
DID (ref = 2019, Time 1)												
2020, Time 2	0.001 (−0.008–0.009)	0.887	0.004 (−0.005–0.013)	0.349	−0.071 (−0.156–0.014)	0.100	0.003 (−0.004–0.009)	0.392	0.009 (−0.003–0.021)	0.147	0.008 (−0.001–0.017)	0.085
2020, Time 3	−0.003 (−0.046–0.039)	0.880	−0.053 (−0.098–0.007)	0.023	−0.118 (−0.496–0.259)	0.538	0.041 (0.014–0.069)	0.003	0.075 (0.016–0.134)	0.013	−0.001 (−0.048–0.047)	0.983

Covariates include age, sex, location, and presence of counselor.

a Insomnia state.

Unemployment was positively and significantly associated with age and absence of counselors in both 2020 and 2019 (Table 2 and Table S5). Household economic decline was positively and significantly associated with age, location outside of Fukushima Prefecture, and absence of counselors in both 2020 and 2019. Absence of counselors was positively and significantly associated with other outcomes (i.e., psychological distress, problem drinking, AIS-SJ, and interpersonal problems) in both 2020 and 2019.

**Table 2:** Association between sociodemographic factors and outcomes in 2020. OR: odds ratio, CI: confidence interval, B: unstandardized regression coefficient (written in *italic*).

	Psychological distress (N = 25153)		Problem drinking (N = 24668)		AIS-SJ^a^ (N = 22991)		Unemployment (N = 24749)		Household economic decline (N = 24750)		Interpersonal problems (N = 24752)	
	OR (95%CI)	P	OR (95%CI)	P	B (95%CI)	P	OR (95%CI)	P	OR (95%CI)	P	OR (95%CI)	P
Age (ref = 15–49)												
50–64	0.652 (0.556–0.766)	<0.001	1.354 (1.155–1.586)	<0.001	*0.280 (0.198–0.362)*	<0.001	1.585 (1.284–1.957)	<0.001	1.448 (1.283–1.635)	<0.001	0.769 (0.668–0.885)	<0.001
65 and more	0.505 (0.441–0.578)	<0.001	0.988 (0.858–1.139)	0.873	*0.272 (0.204–0.341)*	<0.001	1.013 (0.834–1.230)	0.896	1.336 (1.203–1.484)	<0.001	0.453 (0.399–0.513)	<0.001
Sex (ref = male)												
Female	0.991 (0.882–1.114)	0.886	1.011 (0.907–1.127)	0.841	*0.022 (-0.034–0.078)*	0.439	1.004 (0.868–1.163)	0.954	1.010 (0.934–1.094)	0.796	0.957 (0.860–1.065)	0.422
Location (ref = inside of Fukushima Prefecture)												
Outside of Fukushima Prefecture	1.436 (1.240–1.662)	<0.001	0.993 (0.850–1.161)	0.933	*0.350 (0.270–0.430)*	<0.001	0.935 (0.755–1.157)	0.534	1.170 (1.048–1.305)	0.005	0.905 (0.778–1.053)	0.198
Counselor (ref = presence)												
Absence	3.460 (3.040–3.938)	<0.001	1.640 (1.425–1.887)	<0.001	*0.875 (0.790–0.960)*	<0.001	2.283 (1.922–2.712)	<0.001	2.279 (2.067–2.514)	<0.001	2.438 (2.147–2.769)	<0.001
Time (ref = Time 1)												
Time 2	1.097 (0.967–1.244)	0.151	1.119 (0.996–1.257)	0.058	*0.046 (-0.015–0.107)*	0.141	0.998 (0.849–1.173)	0.980	1.113 (1.021–1.213)	0.015	1.092 (0.973–1.225)	0.135
Time 3	1.073 (0.667–1.726)	0.773	0.576 (0.305–1.088)	0.089	*−0.005 (-0.248–0.238)*	0.969	1.676 (0.986–2.850)	0.056	1.751 (1.288–2.381)	<0.001	1.013 (0.651–1.576)	0.956

a Insomnia state.

## 4 Discussion

This study quantified the association of COVID-19 and related public measures such as declaration of the state of emergency with economic status and mental health among the people affected by the Fukushima disaster using the DID method. The number and proportions of participants in each period were almost similar between 2020 and 2019, but the 2018 dataset showed an abundance in Time 2. This is probably due to the difference in the way the questionnaires were distributed: questionnaires were sent to all the participants in 2020 and 2019 by February 14 and 15, respectively, but the distribution in 2018 was done by February 26. Since we regarded the 2019 dataset as the control group to estimate the DID, the difference in the distribution methods in 2018 did not affect the findings. Furthermore, we confirmed that no significant DIDs were found for the outcomes except psychological distress and unemployment through comparisons between 2018 and 2019 (i.e., before the COVID-19 outbreak).

Comparisons between 2020 and 2019 showed significant and positive DIDs for unemployment and household economic decline in Time 3. The significant of DID for unemployment should be interpreted with caution. This is because, we still found a significant DID in 2018 and 2019, even though we used the two years prior to the COVID-19 pandemic. Contrary, for household economic decline, no significant DID was found between 2018 and 2019. Observations of significant DIDs in Time 3 (not in Time 2) indicated that the association of COVID-19 and related public measures with economic status, especially household economic decline, was clear after the declaration of the state of emergency during the COVID-19 pandemic. Sensitivity analysis also showed that in Time 3, there was a significant difference in household economic decline between 2019 and 2020. This was consistent with the data of reports that serious economic losses occurred in Japan after the declaration of the state of emergency (Cabinet Office Japan, 2020).

Contrary to the association with economic status, mental health status did not significantly worsen among the participants. Rather, problem drinking possibly improved after the declaration of the state of emergency, which might reflect the avoidance of eating out. Another possibility is a high level of awareness about problem drinking. In the aftermath of the Fukushima disaster, there was an increase in problem drinking among the affected people (Ueda et al., 2019). Fukushima Prefecture has conducted a series of campaigns to promote moderate drinking since the Fukushima disaster. This could possibly have created a high level of awareness about problem drinking, which might have been effective especially after the declaration of the state of emergency. Maintenance of good health status among the participants can be explained by three reasons. First, the COVID-19 outbreak itself was limited to Fukushima Prefecture, as the number of confirmed cases was only 81 by May 21, 2020 (Fukushima Prefecture, 2020). Second, the durations since public measures such as requests for voluntary stay-at-home and the declaration of the state of emergency were not so long that the participants experienced worsening mental health status. Third, the participants might have unique and resilient characteristics, as they had already experienced the Fukushima disaster (Takebayashi et al., 2020). However, there is an urgent need for careful attention and long-term support for such people because worsening economic status could lead to subsequent decline in health status and increase in suicide (McIntyre & Lee, 2020; Suzuki et al., 2015).

Overall, absence of counselors is a strong risk factor across multivariate outcomes including psychological distress, problem drinking, insomnia state, unemployment, household economic decline, and interpersonal problems. Absence of counselors might lead to less availability of a variety of emotional, informational, instrumental and appraisal support (Langford et al., 1997). In addition, COVID-19 and public measures could increase loneliness (Tull et al., 2020). Hence, suitable measures regarding this can be developed and implemented.

The strength of this study is that it successfully provides novel knowledge on the effect of COVID-19 and relevant public measures using the DID method based on surveys before and during the pandemic. However, this study had some limitations. First, because the response rates were not very high (approximately 20%), there were potential selection biases. A previous study showed that more respondents are unemployed and have less psychological distress compared with non-respondents (Horikoshi et al., 2017). Therefore the prevalence of psychological distress might be underestimated, whereas that of unemployment is overestimated. However, since survey response rates varied by age and sex (Horikoshi et al., 2017), adjustment for such covariates in the multiple regression analyses reduced the selection biases created by these differences. Second, in this study, we corrected for the bias caused by differences in the time of responses by using the difference between 2019 and 2020 and by adjusting for covariates such as age and sex. However, we cannot discard the possibility that the bias has not been completely eliminated. Third, we did not analyze the differences in types of counsellors. Further studies warrant the clarification of associations between specific types of counselors and economic status or mental health. Fourth, because the participants comprised people affected by the Fukushima disaster, the findings may not be expanded to the general population.

## 5 Conclusions

This study reported the association of COVID-19 and relevant public measures with multivariate outcomes (i.e., psychological distress, problem drinking, insomnia state, unemployment, household economic decline, and interpersonal problems) among people affected by the Fukushima disaster using the DID method. While economic status, especially household economic decline, worsened after the declaration of the state of emergency, mental health was maintained by May 21, 2020. Since worsening economic status could subsequently reduce mental health status and absence of counselors is a common risk factor for multivariate outcomes, identifying people who do not have counselors and providing support to them can help prevent subsequent mental health decline in these patients.

## Financial support

This survey was supported by the national ‘Health Fund for Children and Adults Affected by the Nuclear Incident’.

## Ethics statement

Ethical approval for this study was granted by the Fukushima Medical University Ethics Committee (approval number: 2148).

## Declaration of competing interest

None.

## CRediT authorship contribution statement

**Michio Murakami:** Conceptualization, Formal analysis, Methodology, Visualization, Writing – original draft. **Tomoyuki Kobayashi:** Formal analysis, Methodology, Writing – review & editing. **Yuichi Oikawa:** Writing – review & editing. **Saori Goto:** Writing – review & editing. **Maho Momoi:** Writing – review & editing. **Yoshitake Takebayashi:** Writing – review & editing. **Tetsuya Ohira:** Writing – review & editing. **Seiji Yasumura:** Writing – review & editing. **Masaharu Maeda:** Conceptualization, Writing – review & editing.
