# Supplementary material for: Associations of the COVID-19 pandemic with the economic status and mental health of people affected by the Fukushima disaster using the difference-in-differences method: The Fukushima Health Management Survey
Source: SSM Popul Health. 2021 Apr 15;14:100801. doi: 10.1016/j.ssmph.2021.100801 (PMC8095183; doi:10.1016/j.ssmph.2021.100801)
Supplement: Supplementary file 1 — Multimedia component 1 [file mmc1.docx]

Fig. S1. Cumulative number of participants in each survey.

A: February 26, 2020, voluntary stay-at-home requested by the central government.

B: April 16, 2020, declaration of the state of emergency in all prefectures, including Fukushima Prefecture.

Table S1. Dates of governmental public measures regarding voluntary stay-at-home requests and declaration of the state of emergency.

| Dates | Action taken |
| --- | --- |
| February 26, 2020 | Voluntary stay-at-home requests. |
| March 10, 2020 | Re-voluntary stay-at-home requests. |
| April 7, 2020 | Declaration of the state of emergency in seven prefectures (Saitama, Chiba, Tokyo, Kanagawa, Osaka, Hyogo and Fukuoka) |
| April 16, 2020 | Declaration of the state of emergency in all prefectures including Fukushima Prefecture. |
| May 14, 2020 | Shrinkage of declaration of the state of emergency into eight prefectures (Hokkaido, Saitama, Chiba, Tokyo, Kanagawa, Kyoto, Osaka, and Hyogo). |
| May 21, 2020 | Shrinkage of declaration of the state of emergency into five prefectures (Hokkaido, Saitama, Chiba, Tokyo, and Kanagawa). |
| May 25, 2020 | Lift of the state of emergency in all prefectures. |

Table S2. Characteristics of participants. SD: standard deviation.

|  |  | Time 1 | Time 2 | Time 3 | Total |
| --- | --- | --- | --- | --- | --- |
| Total |  |  |  |  |  |
| 2018 | - | 9416 | 22068 | 820 | 32304 |
| 2019 | - | 20577 | 11121 | 242 | 31940 |
| 2020 | - | 21112 | 9031 | 384 | 30527 |
| Age |  |  |  |  |  |
| 2018 | 15–49 | 2349 (24.9%) | 4586 (20.8%) | 333 (40.6%) | 7268 (22.5%) |
|  | 50–64 | 2050 (21.8%) | 5025 (22.8%) | 225 (27.4%) | 7300 (22.6%) |
|  | 65 and more | 5017 (53.3%) | 12457 (56.4%) | 262 (32.0%) | 17736 (54.9%) |
| 2019 | 15–49 | 4076 (19.8%) | 2661 (23.9%) | 124 (51.2%) | 6861 (21.5%) |
|  | 50–64 | 4271 (20.8%) | 2438 (21.9%) | 61 (25.2%) | 6770 (21.2%) |
|  | 65 and more | 12230 (59.4%) | 6022 (54.1%) | 57 (23.6%) | 18309 (57.3%) |
| 2020 | 15–49 | 3945 (18.7%) | 2169 (24.0%) | 195 (50.8%) | 6309 (20.7%) |
|  | 50–64 | 4156 (19.7%) | 1845 (20.4%) | 93 (24.2%) | 6094 (20.0%) |
|  | 65 and more | 13011 (61.6%) | 5017 (55.6%) | 96 (25.0%) | 18124 (59.4%) |
| Sex |  |  |  |  |  |
| 2018 | Male | 4383 (46.5%) | 9799 (44.4%) | 345 (42.1%) | 14527 (45.0%) |
|  | Female | 5033 (53.5%) | 12269 (55.6%) | 475 (57.9%) | 17777 (55.0%) |
| 2019 | Male | 9575 (46.5%) | 4799 (43.2%) | 104 (43.0%) | 14478 (45.3%) |
|  | Female | 11002 (53.5%) | 6322 (56.8%) | 138 (57.0%) | 17462 (54.7%) |
| 2020 | Male | 9957 (47.2%) | 4010 (44.4%) | 159 (41.4%) | 14126 (46.3%) |
|  | Female | 11155 (52.8%) | 5021 (55.6%) | 225 (58.6%) | 16401 (53.7%) |
| Location |  |  |  |  |  |
| 2018 | Inside of Fukushima Prefecture | 8064 (85.6%) | 18967 (85.9%) | 682 (83.2%) | 27713 (85.8%) |
|  | Outside of Fukushima Prefecture | 1352 (14.4%) | 3101 (14.1%) | 138 (16.8%) | 4591 (14.2%) |
| 2019 | Inside of Fukushima Prefecture | 17747 (86.2%) | 9655 (86.8%) | 198 (81.8%) | 27600 (86.4%) |
|  | Outside of Fukushima Prefecture | 2830 (13.8%) | 1466 (13.2%) | 44 (18.2%) | 4340 (13.6%) |
| 2020 | Inside of Fukushima Prefecture | 18390 (87.1%) | 7710 (85.4%) | 317 (82.6%) | 26417 (86.5%) |
|  | Outside of Fukushima Prefecture | 2722 (12.9%) | 1321 (14.6%) | 67 (17.4%) | 4110 (13.5%) |
| Counselor |  |  |  |  |  |
| 2018 | Presence | 6924 (85.3%) | 16002 (86.6%) | 607 (85.6%) | 23533 (86.2%) |
|  | Absence | 1194 (14.7%) | 2468 (13.4%) | 102 (14.4%) | 3764 (13.8%) |
| 2019 | Presence | 14864 (86.6%) | 7968 (86.9%) | 172 (86.4%) | 23004 (86.7%) |
|  | Absence | 2294 (13.4%) | 1201 (13.1%) | 27 (13.6%) | 3522 (13.3%) |
| 2020 | Presence | 15774 (87.3%) | 6702 (87.4%) | 312 (92.6%) | 22788 (87.4%) |
|  | Absence | 2294 (12.7%) | 965 (12.6%) | 25 (7.4%) | 3284 (12.6%) |
| K6 |  |  |  |  |  |
| 2018 | <13 | 8551 (94.5%) | 19409 (93.3%) | 746 (93.6%) | 28706 (93.6%) |
|  | 13 and more | 500 (5.5%) | 1403 (6.7%) | 51 (6.4%) | 1954 (6.4%) |
| 2019 | <13 | 18608 (94.5%) | 9837 (94.0%) | 215 (93.1%) | 28660 (94.3%) |
|  | 13 and more | 1077 (5.5%) | 630 (6.0%) | 16 (6.9%) | 1723 (5.7%) |
| 2020 | <13 | 19181 (95.4%) | 8060 (94.6%) | 351 (93.1%) | 27592 (95.1%) |
|  | 13 and more | 931 (4.6%) | 457 (5.4%) | 26 (6.9%) | 1414 (4.9%) |
| CAGE |  |  |  |  |  |
| 2018 | <2 | 8350 (94.0%) | 19333 (94.4%) | 727 (94.2%) | 28410 (94.2%) |
|  | 2 and more | 537 (6.0%) | 1152 (5.6%) | 45 (5.8%) | 1734 (5.8%) |
| 2019 | <2 | 18175 (94.2%) | 9642 (94.2%) | 200 (90.5%) | 28017 (94.1%) |
|  | 2 and more | 1127 (5.8%) | 594 (5.8%) | 21 (9.5%) | 1742 (5.9%) |
| 2020 | <2 | 18764 (94.4%) | 7845 (93.9%) | 335 (96.0%) | 26944 (94.3%) |
|  | 2 and more | 1109 (5.6%) | 508 (6.1%) | 14 (4.0%) | 1631 (5.7%) |
| AIS-SJ |  |  |  |  |  |
| 2018 | Mean (SD) [N] | 2.68 (2.20) [8062] | 2.86 (2.25) [18280] | 2.81 (2.26) [716] | 2.80 (2.24) [27058] |
| 2019 | Mean (SD) [N] | 2.69 (2.20) [17567] | 2.80 (2.21) [9273] | 2.79 (2.13) [219] | 2.73 (2.20) [27059] |
| 2020 | Mean (SD) [N] | 2.65 (2.16) [18285] | 2.71 (2.21) [7673] | 2.60 (2.27) [345] | 2.67 (2.18) [26303] |
| Unemployment |  |  |  |  |  |
| 2018 | Absence | 8245 (95.9%) | 19077 (95.6%) | 719 (95.5%) | 28041 (95.7%) |
|  | Presence | 351 (4.1%) | 887 (4.4%) | 34 (4.5%) | 1272 (4.3%) |
| 2019 | Absence | 18750 (96.6%) | 10094 (96.7%) | 225 (98.7%) | 29069 (96.7%) |
|  | Presence | 655 (3.4%) | 341 (3.3%) | 3 (1.3%) | 999 (3.3%) |
| 2020 | Absence | 19220 (97.0%) | 8158 (96.9%) | 345 (95.6%) | 27723 (96.9%) |
|  | Presence | 602 (3.0%) | 261 (3.1%) | 16 (4.4%) | 879 (3.1%) |
| Household economic decline |  |  |  |  |  |
| 2018 | Absence | 7501 (87.2%) | 17488 (87.6%) | 650 (86.3%) | 25639 (87.5%) |
|  | Presence | 1098 (12.8%) | 2473 (12.4%) | 103 (13.7%) | 3674 (12.5%) |
| 2019 | Absence | 17035 (87.8%) | 9121 (87.4%) | 203 (89.0%) | 26359 (87.7%) |
|  | Presence | 2373 (12.2%) | 1313 (12.6%) | 25 (11.0%) | 3711 (12.3%) |
| 2020 | Absence | 17621 (88.9%) | 7414 (88.1%) | 305 (84.5%) | 25340 (88.6%) |
|  | Presence | 2200 (11.1%) | 1006 (11.9%) | 56 (15.5%) | 3262 (11.4%) |
| Interpersonal problems |  |  |  |  |  |
| 2018 | Absence | 7984 (92.8%) | 18567 (93.1%) | 698 (92.6%) | 27249 (93.0%) |
|  | Presence | 615 (7.2%) | 1380 (6.9%) | 56 (7.4%) | 2051 (7.0%) |
| 2019 | Absence | 18202 (93.8%) | 9788 (93.8%) | 212 (93.0%) | 28202 (93.8%) |
|  | Presence | 1202 (6.2%) | 647 (6.2%) | 16 (7.0%) | 1865 (6.2%) |
| 2020 | Absence | 18698 (94.3%) | 7879 (93.6%) | 336 (93.3%) | 26913 (94.1%) |
|  | Presence | 1123 (5.7%) | 541 (6.4%) | 24 (6.7%) | 1688 (5.9%) |

Table S3. Associations between variables other than difference-in-differences and outcomes (adjusted by covariates). (a) 2018 vs. 2019, (b) 2020 vs. 2019. B: unstandardized regression coefficient, CI: confidence interval.

|  | Psychological distress | | Problem drinking | | AIS-SJ^a^ | | Unemployment | | Household economic decline | | Interpersonal problems | |
| --- | --- | --- | --- | --- | --- | --- | --- | --- | --- | --- | --- | --- |
|  | B (95%CI) | P | B (95%CI) | P | B (95%CI) | P | B (95%CI) | P | B (95%CI) | P | B (95%CI) | P |
| (a) 2018 vs 2019 |  |  |  |  |  |  |  |  |  |  |  |  |
| Year (ref=2019) |  |  |  |  |  |  |  |  |  |  |  |  |
| 2018 | -0.001 (-0.007–0.005) | 0.808 | 0.003 (-0.003–0.010) | 0.301 | 0.011 (-0.049–0.072) | 0.712 | 0.007 (0.002–0.012) | 0.012 | 0.005 (-0.004–0.014) | 0.304 | 0.007 (0.000–0.014) | 0.042 |
| Time (ref=Time 1) |  |  |  |  |  |  |  |  |  |  |  |  |
| Time 2 | 0.004 (-0.002–0.009) | 0.232 | 0.004 (-0.002–0.010) | 0.231 | 0.117 (0.058–0.175) | <0.001 | -0.002 (-0.007–0.002) | 0.315 | 0.002 (-0.006–0.011) | 0.585 | -0.003 (-0.010–0.003) | 0.281 |
| Time 3 | 0.008 (-0.027–0.043) | 0.650 | 0.028 (-0.012–0.068) | 0.170 | 0.138 (-0.146–0.422) | 0.341 | -0.022 (-0.037–-0.007) | 0.005 | -0.012 (-0.055–0.031) | 0.586 | -0.001 (-0.040–0.037) | 0.953 |
| Age (ref=15–49) |  |  |  |  |  |  |  |  |  |  |  |  |
| 50–64 | -0.017 (-0.023–-0.011) | <0.001 | 0.004 (-0.002–0.010) | 0.245 | 0.386 (0.331–0.441) | <0.001 | 0.021 (0.016–0.026) | <0.001 | 0.035 (0.027–0.043) | <0.001 | -0.017 (-0.024–-0.010) | <0.001 |
| 65 and more | -0.027 (-0.032–-0.022) | <0.001 | -0.017 (-0.022–-0.012) | <0.001 | 0.387 (0.340–0.434) | <0.001 | 0.002 (-0.002–0.006) | 0.276 | 0.027 (0.020–0.034) | <0.001 | -0.049 (-0.055–-0.043) | <0.001 |
| Sex (ref=male) |  |  |  |  |  |  |  |  |  |  |  |  |
| Female | 0.015 (0.011–0.019) | <0.001 | -0.080 (-0.085–-0.076) | <0.001 | 0.321 (0.281–0.361) | <0.001 | -0.017 (-0.021–-0.014) | <0.001 | -0.022 (-0.028–-0.016) | <0.001 | 0.007 (0.002–0.011) | 0.002 |
| Location (ref=inside of Fukushima Prefecture) |  |  |  |  |  |  |  |  |  |  |  |  |
| Outside of Fukushima Prefecture | 0.019 (0.012–0.025) | <0.001 | 0.002 (-0.004–0.008) | 0.445 | 0.350 (0.293–0.407) | <0.001 | -0.004 (-0.008–0.001) | 0.106 | 0.019 (0.011–0.028) | <0.001 | -0.004 (-0.010–0.003) | 0.271 |
| Counselor (ref=presence) |  |  |  |  |  |  |  |  |  |  |  |  |
| Absence | 0.109 (0.100–0.118) | <0.001 | 0.022 (0.015–0.030) | <0.001 | 1.064 (1.000–1.128) | <0.001 | 0.030 (0.024–0.036) | <0.001 | 0.102 (0.091–0.112) | <0.001 | 0.072 (0.063–0.080) | <0.001 |
|  |  |  |  |  |  |  |  |  |  |  |  |  |
| (b) 2020 vs 2019 |  |  |  |  |  |  |  |  |  |  |  |  |
| Year (ref=2019) |  |  |  |  |  |  |  |  |  |  |  |  |
| 2020 | -0.005 (-0.010–-0.001) | 0.019 | -0.001 (-0.006–0.004) | 0.753 | -0.008 (-0.056–0.040) | 0.746 | -0.004 (-0.007–0.000) | 0.062 | -0.011 (-0.018–-0.004) | 0.001 | -0.005 (-0.010–0.000) | 0.042 |
| Time (ref=Time 1) |  |  |  |  |  |  |  |  |  |  |  |  |
| Time 2 | 0.004 (-0.002–0.010) | 0.223 | 0.003 (-0.003–0.009) | 0.356 | 0.117 (0.058–0.176) | <0.001 | -0.003 (-0.007–0.002) | 0.251 | 0.002 (-0.007–0.010) | 0.665 | -0.003 (-0.009–0.003) | 0.333 |
| Time 3 | 0.008 (-0.027–0.042) | 0.670 | 0.030 (-0.011–0.070) | 0.151 | 0.130 (-0.155–0.414) | 0.371 | -0.023 (-0.038–-0.008) | 0.003 | -0.013 (-0.056–0.030) | 0.543 | 0.001 (-0.038–0.039) | 0.977 |
| Age (ref=15–49) |  |  |  |  |  |  |  |  |  |  |  |  |
| 50–64 | -0.019 (-0.025–-0.013) | <0.001 | 0.013 (0.006–0.019) | <0.001 | 0.347 (0.290–0.403) | <0.001 | 0.016 (0.011–0.021) | <0.001 | 0.034 (0.025–0.042) | <0.001 | -0.015 (-0.022–-0.008) | <0.001 |
| 65 and more | -0.030 (-0.035–-0.025) | <0.001 | -0.007 (-0.012–-0.002) | 0.009 | 0.329 (0.282–0.377) | <0.001 | 0.001 (-0.003–0.004) | 0.659 | 0.025 (0.019–0.032) | <0.001 | -0.045 (-0.050–-0.039) | <0.001 |
| Sex (ref=male) |  |  |  |  |  |  |  |  |  |  |  |  |
| Female | 0.006 (0.002–0.009) | 0.004 | -0.040 (-0.044–-0.036) | <0.001 | 0.160 (0.121–0.199) | <0.001 | -0.007 (-0.010–-0.004) | <0.001 | -0.010 (-0.016–-0.005) | <0.001 | 0.003 (-0.001–0.007) | 0.180 |
| Location (ref=inside of Fukushima Prefecture) |  |  |  |  |  |  |  |  |  |  |  |  |
| Outside of Fukushima Prefecture | 0.021 (0.014–0.027) | <0.001 | -0.004 (-0.010–0.002) | 0.202 | 0.340 (0.283–0.398) | <0.001 | -0.001 (-0.005–0.004) | 0.739 | 0.019 (0.010–0.027) | <0.001 | -0.004 (-0.010–0.003) | 0.260 |
| Counselor (ref=presence) |  |  |  |  |  |  |  |  |  |  |  |  |
| Absence | 0.092 (0.083–0.100) | <0.001 | 0.029 (0.022–0.037) | <0.001 | 0.911 (0.846–0.976) | <0.001 | 0.032 (0.026–0.038) | <0.001 | 0.106 (0.096–0.117) | <0.001 | 0.064 (0.056–0.073) | <0.001 |

^a^ insomnia state.

Table S4. Associations between variables and outcomes (time data were classified into six categories). (a) 2018 vs. 2019, (b) 2020 vs. 2019. B: unstandardized regression coefficient, CI: confidence interval.

|  | Psychological distress | | Problem drinking | | AIS-SJ^a^ | | Unemployment | | Household economic decline | | Interpersonal problems | |
| --- | --- | --- | --- | --- | --- | --- | --- | --- | --- | --- | --- | --- |
|  | B (95%CI) | P | B (95%CI) | P | B (95%CI) | P | B (95%CI) | P | B (95%CI) | P | B (95%CI) | P |
| (a) 2018 vs 2019 |  |  |  |  |  |  |  |  |  |  |  |  |
| Year (ref=2019 Time3) |  |  |  |  |  |  |  |  |  |  |  |  |
| 2018 Time 1 | -0.009 (-0.043–0.026) | 0.622 | -0.025 (-0.065–0.016) | 0.229 | -0.127 (-0.413–0.160) | 0.386 | 0.029 (0.013–0.044) | <0.001 | 0.017 (-0.027–0.060) | 0.450 | 0.008 (-0.030–0.047) | 0.671 |
| 2018 Time 2 | 0.005 (-0.030–0.039) | 0.798 | -0.028 (-0.068–0.012) | 0.173 | 0.041 (-0.243–0.325) | 0.778 | 0.031 (0.016–0.046) | <0.001 | 0.012 (-0.031–0.055) | 0.587 | 0.007 (-0.032–0.046) | 0.724 |
| 2018 Time 3 | -0.006 (-0.044–0.033) | 0.771 | -0.031 (-0.075–0.012) | 0.155 | 0.011 (-0.317–0.339) | 0.947 | 0.032 (0.010–0.054) | 0.004 | 0.029 (-0.021–0.079) | 0.250 | 0.001 (-0.042–0.045) | 0.948 |
| 2019 Time 1 | -0.008 (-0.043–0.027) | 0.650 | -0.028 (-0.068–0.012) | 0.170 | -0.138 (-0.422–0.146) | 0.341 | 0.022 (0.007–0.037) | 0.005 | 0.012 (-0.031–0.055) | 0.586 | 0.001 (-0.037–0.040) | 0.953 |
| 2019 Time 2 | -0.004 (-0.039–0.030) | 0.803 | -0.024 (-0.065–0.016) | 0.235 | -0.021 (-0.307–0.264) | 0.883 | 0.019 (0.004–0.035) | 0.013 | 0.014 (-0.029–0.057) | 0.516 | -0.002 (-0.041–0.036) | 0.908 |
| Age (ref=15–49) |  |  |  |  |  |  |  |  |  |  |  |  |
| 50–64 | -0.017 (-0.023–-0.011) | <0.001 | 0.004 (-0.002–0.010) | 0.245 | 0.386 (0.331–0.441) | <0.001 | 0.021 (0.016–0.026) | <0.001 | 0.035 (0.027–0.043) | <0.001 | -0.017 (-0.024–-0.010) | <0.001 |
| 65 and more | -0.027 (-0.032–-0.022) | <0.001 | -0.017 (-0.022–-0.012) | <0.001 | 0.387 (0.340–0.434) | <0.001 | 0.002 (-0.002–0.006) | 0.276 | 0.027 (0.020–0.034) | <0.001 | -0.049 (-0.055–-0.043) | <0.001 |
| Sex (ref=male) |  |  |  |  |  |  |  |  |  |  |  |  |
| Female | 0.015 (0.011–0.019) | <0.001 | -0.080 (-0.085–-0.076) | <0.001 | 0.321 (0.281–0.361) | <0.001 | -0.017 (-0.021–-0.014) | <0.001 | -0.022 (-0.028–-0.016) | <0.001 | 0.007 (0.002–0.011) | 0.002 |
| Location (ref=inside of Fukushima Prefecture) |  |  |  |  |  |  |  |  |  |  |  |  |
| Outside of Fukushima Prefecture | 0.019 (0.012–0.025) | <0.001 | 0.002 (-0.004–0.008) | 0.445 | 0.350 (0.293–0.407) | <0.001 | -0.004 (-0.008–0.001) | 0.106 | 0.019 (0.011–0.028) | <0.001 | -0.004 (-0.010–0.003) | 0.271 |
| Counselor (ref=presence) |  |  |  |  |  |  |  |  |  |  |  |  |
| Absence | 0.109 (0.100–0.118) | <0.001 | 0.022 (0.015–0.030) | <0.001 | 1.064 (1.000–1.128) | <0.001 | 0.030 (0.024–0.036) | <0.001 | 0.102 (0.091–0.112) | <0.001 | 0.072 (0.063–0.080) | <0.001 |
| (b) 2020 vs 2019 |  |  |  |  |  |  |  |  |  |  |  |  |
| Year (ref=2019 Time3) |  |  |  |  |  |  |  |  |  |  |  |  |
| 2019 Time 1 | -0.008 (-0.042–0.027) | 0.670 | -0.030 (-0.070–0.011) | 0.151 | -0.130 (-0.414–0.155) | 0.371 | 0.023 (0.008–0.038) | 0.003 | 0.013 (-0.030–0.056) | 0.543 | -0.001 (-0.039–0.038) | 0.977 |
| 2019 Time 2 | -0.004 (-0.039–0.031) | 0.826 | -0.027 (-0.068–0.014) | 0.195 | -0.013 (-0.299–0.273) | 0.930 | 0.020 (0.005–0.035) | 0.010 | 0.015 (-0.028–0.058) | 0.490 | -0.004 (-0.042–0.035) | 0.853 |
| 2020 Time 1 | -0.013 (-0.048–0.022) | 0.461 | -0.031 (-0.071–0.010) | 0.140 | -0.138 (-0.422–0.147) | 0.342 | 0.019 (0.004–0.034) | 0.013 | 0.002 (-0.041–0.045) | 0.921 | -0.006 (-0.044–0.033) | 0.768 |
| 2020 Time 2 | -0.009 (-0.044–0.026) | 0.622 | -0.023 (-0.064–0.017) | 0.261 | -0.092 (-0.379–0.195) | 0.529 | 0.019 (0.004–0.035) | 0.013 | 0.013 (-0.030–0.056) | 0.553 | -0.001 (-0.040–0.038) | 0.962 |
| 2020 Time 3 | -0.009 (-0.051–0.034) | 0.685 | -0.053 (-0.098–-0.008) | 0.020 | -0.126 (-0.500–0.248) | 0.508 | 0.038 (0.010–0.065) | 0.007 | 0.064 (0.005–0.122) | 0.032 | -0.006 (-0.053–0.041) | 0.811 |
| Age (ref=15–49) |  |  |  |  |  |  |  |  |  |  |  |  |
| 50–64 | -0.019 (-0.025–-0.013) | <0.001 | 0.013 (0.006–0.019) | <0.001 | 0.347 (0.290–0.403) | <0.001 | 0.016 (0.011–0.021) | <0.001 | 0.034 (0.025–0.042) | <0.001 | -0.015 (-0.022–-0.008) | <0.001 |
| 65 and more | -0.030 (-0.035–-0.025) | <0.001 | -0.007 (-0.012–-0.002) | 0.009 | 0.329 (0.282–0.377) | <0.001 | 0.001 (-0.003–0.004) | 0.659 | 0.025 (0.019–0.032) | <0.001 | -0.045 (-0.050–-0.039) | <0.001 |
| Sex (ref=male) |  |  |  |  |  |  |  |  |  |  |  |  |
| Female | 0.006 (0.002–0.009) | 0.004 | -0.040 (-0.044–-0.036) | <0.001 | 0.160 (0.121–0.199) | <0.001 | -0.007 (-0.010–-0.004) | <0.001 | -0.010 (-0.016–-0.005) | <0.001 | 0.003 (-0.001–0.007) | 0.180 |
| Location (ref=inside of Fukushima Prefecture) |  |  |  |  |  |  |  |  |  |  |  |  |
| Outside of Fukushima Prefecture | 0.021 (0.014–0.027) | <0.001 | -0.004 (-0.010–0.002) | 0.202 | 0.340 (0.283–0.398) | <0.001 | -0.001 (-0.005–0.004) | 0.739 | 0.019 (0.010–0.027) | <0.001 | -0.004 (-0.010–0.003) | 0.260 |
| Counselor (ref=presence) |  |  |  |  |  |  |  |  |  |  |  |  |
| Absence | 0.092 (0.083–0.100) | <0.001 | 0.029 (0.022–0.037) | <0.001 | 0.911 (0.846–0.976) | <0.001 | 0.032 (0.026–0.038) | <0.001 | 0.106 (0.096–0.117) | <0.001 | 0.064 (0.056–0.073) | <0.001 |

^a^ insomnia state.

Table S5. Association between sociodemographic factors and outcomes in 2019. OR: odds ratio, CI: confidence interval, B: unstandardized regression coefficient (written in *italic*).

|  | Psychological distress (N=25593) | | Problem drinking  (N=24992) | | AIS-SJ^a^  (N=23021) | | Unemployment  (N=25316) | | Household economic decline (N=25320) | | Interpersonal problems  (N=25314) | |
| --- | --- | --- | --- | --- | --- | --- | --- | --- | --- | --- | --- | --- |
|  | OR (95%CI) | P | OR (95%CI) | P | B (95%CI) | P | OR (95%CI) | P | OR (95%CI) | P | OR (95%CI) | P |
| Age (ref=15–49) |  |  |  |  |  |  |  |  |  |  |  |  |
| 50–64 | 0.782 (0.673–0.907) | 0.001 | 1.058 (0.908–1.234) | 0.468 | *0.420 (0.340–0.500)* | <0.001 | 1.592 (1.302–1.946) | <0.001 | 1.356 (1.210–1.519) | <0.001 | 0.849 (0.743–0.970) | 0.016 |
| 65 and more | 0.616 (0.541–0.701) | <0.001 | 0.718 (0.624–0.825) | <0.001 | *0.402 (0.333–0.470)* | <0.001 | 1.037 (0.860–1.249) | 0.706 | 1.257 (1.138–1.388) | <0.001 | 0.487 (0.430–0.550) | <0.001 |
| Sex (ref=male) |  |  |  |  |  |  |  |  |  |  |  |  |
| Female | 1.309 (1.169–1.466) | <0.001 | 0.174 (0.152–0.200) | <0.001 | *0.311 (0.254–0.367)* | <0.001 | 0.636 (0.551–0.734) | <0.001 | 0.815 (0.755–0.881) | <0.001 | 1.161 (1.045–1.290) | 0.005 |
| Location (ref=inside of Fukushima Prefecture) |  |  |  |  |  |  |  |  |  |  |  |  |
| Outside of Fukushima Prefecture | 1.425 (1.242–1.635) | <0.001 | 0.893 (0.759–1.052) | 0.175 | *0.327 (0.248–0.407)* | <0.001 | 1.018 (0.834–1.242) | 0.861 | 1.210 (1.090–1.344) | <0.001 | 0.979 (0.850–1.128) | 0.770 |
| Counselor (ref=presence) |  |  |  |  |  |  |  |  |  |  |  |  |
| Absence | 4.088 (3.622–4.614) | <0.001 | 1.297 (1.130–1.489) | <0.001 | *0.978 (0.895–1.062)* | <0.001 | 2.068 (1.755–2.438) | <0.001 | 2.217 (2.018–2.435) | <0.001 | 2.454 (2.169–2.776) | <0.001 |
| Time (ref= Time 1) |  |  |  |  |  |  |  |  |  |  |  |  |
| Time 2 | 1.070 (0.954–1.199) | 0.248 | 1.066 (0.951–1.196) | 0.272 | *0.117 (0.058–0.176)* | <0.001 | 0.924 (0.797–1.072) | 0.298 | 1.021 (0.943–1.106) | 0.607 | 0.944 (0.847–1.052) | 0.297 |
| Time 3 | 1.155 (0.660–2.019) | 0.614 | 1.524 (0.893–2.600) | 0.122 | *0.145 (-0.167–0.456)* | 0.363 | 0.307 (0.076–1.245) | 0.098 | 0.865 (0.534–1.399) | 0.553 | 1.004 (0.587–1.716) | 0.989 |

^a^ insomnia state.
